# ﻿Lectotypification, epitypification and history of the name *Plagiotheciumneglectum* Mönk. (Plagiotheciaceae)

**DOI:** 10.3897/phytokeys.189.77839

**Published:** 2022-01-24

**Authors:** Grzegorz J. Wolski, Jarosław Proćków

**Affiliations:** 1 Department of Geobotany and Plant Ecology, Faculty of Biology and Environmental Protection, University of Lodz, ul. Banacha 12/16, 90–237 Lodz, Poland University of Lodz Lodz Poland; 2 Department of Plant Biology, Institute of Environmental Biology, Wrocław University of Environmental and Life Sciences, ul. Kożuchowska 7a, 51–631 Wrocław, Poland Wrocław University of Environmental and Life Sciences Wrocław Poland

**Keywords:** Herbarium B, Herbarium HBG, iconotypus, Mönkemeyer collection, *Orthophyllum* section, synonym, typification

## Abstract

In the protologue of *Plagiotheciumneglectum*, Mönkemeyer (1927) does not indicate any herbarium specimen as a type. The author only gave a short description and attached a figure illustrating selected features of this taxon. The original materials from the Mönkemeyer collection were deposited in the HBG herbarium; however, it is not currently possible to determine their location. Furthermore, one specimen of *P.neglectum*, currently known from the original Mönkemeyer collections, was found in the Herbarium B (B 30 0105646). The features given in the diagnosis of this taxon are consistent with those of the lectotype of *Stereodonnemoralis* Mitt.; only the leaf apex from Figure 207c (Mönkemeyer 1927) is different and thus suggests mixed material. According to Art. 9.1 of the *Shenzhen Code*, Figure 207c represents a holotype of *P.neglectum*. However, due to differences in the leaf apex and according to Art. 9.3 of the *Shenzhen Code*, the part representing the apex should be excluded from the holotype, and the remainder of Figure 207c is consequently designated as a lectotype of the name *P.neglectum*. However, because the lectotype does not include a complete set of significant distinguishing features, an epitype (B 30 0105646) was designated.

## ﻿Introduction

*Plagiotheciumneglectum* Mönk. was described by Mönkemeyer in *Die Laubmoose Europas* (Mönkemeyer 1927). The name was created for specimens previously known as *P.sylvaticumauct*. *non* (Brid.) Bruch & Schimp. (= *P.nemorale* (Mitt.) A.Jaeger), wherein the author also retains and uses the name *P.silvaticum* (*nom. illeg. orthogr. pro P.sylvaticum* (Brid.) Bruch & Schimp.) which is now understood as P.denticulatumvar.obtusifolium (Turner) Moore (Persson 1952; Iwatsuki 1970).

In the diagnosis, Mönkemeyer (1927) did not indicate any herbarium specimen as a type; the only original element is Figure 207c. The taxon is characterized, e.g., by shrunken turf in a dry condition, dense foliage, strong costae, and a very loose areolation of cells. In addition, Mönkemeyer (1927) compared this species with *P.silvaticum* and *P.succulentum* (Wilson) Lindb., also describing that the cell areolation in *P.neglectum* is as wide as that of the former, and the appearance of the turf and the leaves and habitat are similar to the latter.

For decades, *P.neglectum* was treated as a separate species (Jedlička 1948, 1950, 1961; Podpěra 1954; Iwatsuki 1963), as a synonym of *P.silvaticum* (*P.sylvaticum*) (Jensen 1939; Nyholm 1965; Ireland 1969), or a variety of this species – P.silvaticumvar.neglectum (Mönk.) F.Koppe (Koppe 1949; Barkman 1957). Iwatsuki (1970), while examining the lectotype of *Hypnumsylvaticum* Brid., pointed out that this specimen is identical to *P.denticulatum* (Hedw.) Schimp. This researcher also suggested that the plants previously named *P.sylvaticum* and *P.neglectum* should instead be assigned the earlier available epithet, *P.nemorale* (Mitt.) A.Jaeger.

Nyholm (1965) and Ireland (1969), who proposed a synonymization of *P.neglectum* with *P.silvaticum* (*P.sylvaticum*), did not analyze the original materials of this taxon. Also, while Iwatsuki (1970) did not cite herbarium specimens (original material) of *P.neglectum*, his proposed synonymization of this taxon with *P.nemorale* has nevertheless been widely accepted (e.g., Lewinsky 1974; Koponen et al. 1977; Gangulee 1980; Corley et al. 1981; Iwatsuki 1991, 2004; Suzuki 2016; Wolski 2020), and although this proposal was based only on Figure 207c (Mönkemeyer 1927), it was made correctly as this figure is listed in the protologue only.

Considering the above facts, efforts have been made to revise the original herbarium materials of *P.neglectum* from the collection of Wilhelm Mönkemeyer to confirm the correctness of the previous synonymization, which was made in the absence of herbarium specimens.

## ﻿Materials and methods

Before starting the research, efforts were made to find all herbaria in which W. Mönkemeyer collections are deposited. Sayre (1977) pointed out that his materials are deposited in the HBG, H-BR, and S herbaria. Additionally, an analysis of the Index of Botanists (https://kiki.huh.harvard.edu, accessed 15^th^ of May 2021) found some of his herbarium specimens to be stored in the B, H, M and MANCH herbaria.

To find the original materials of *P.neglectum*, contact was made with the staff of all the above-mentioned institutions. All curators indicated that the original materials of the analyzed taxon had been deposited at the Herbarium HBG. This was also confirmed in a paper by Walter and Martienssen (1976) describing the bryological collection of this herbarium.

## ﻿Results

**Figure 1.**
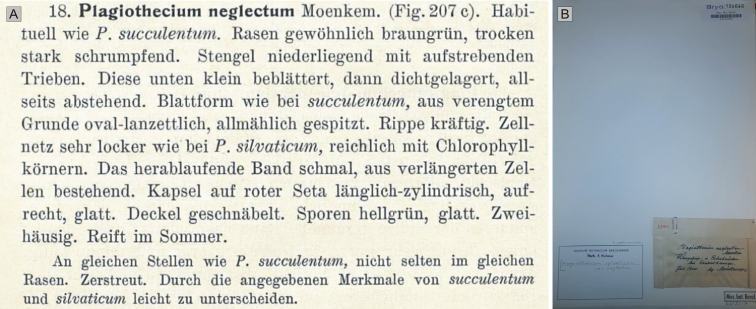
**A** Diagnosis and specimen from the original Mönkemeyer collection. Diagnosis of *P.neglectum* (Mönkemeyer 1927; changed) (accessed from http://bjbdigital.com; 18 October 2021) **B** sheet with the specimen (B 30 0105646) stored in the Berlin herbarium B.

**Figure 2.**
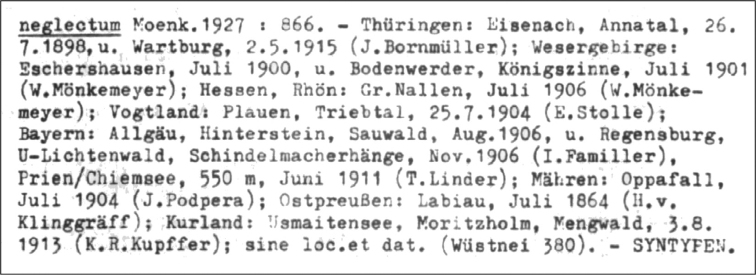
Fragment of the specimen list of the bryological collection of HBG concerning *P.neglectum* (Walter and Martienssen 1976).

In the diagnosis, Mönkemeyer (1927) did not designate any specimen or collection as a type, or identify any original material or even geographical locations of the collection sites of the analyzed specimens. He added only a figure (207c) to the description of the taxon (Fig. 1A). An analysis of the bryological collection of the HBG herbarium (Walter and Martienssen 1976) revealed the presence of 13 “syntypes” of *P.neglectum* (Fig. 2); however, these specimens are incorrectly defined as syntypes because none is cited in the protologue *P.neglectum* (Mönkemeyer 1927). Nevertheless, based on contact with the curator of the bryological collection in the HBG herbarium, it was found that the original material of this taxon has been lost and their location is currently unknown and cannot be determined (Herbarium staff, pers. comm.).

Nevertheless, our research yielded only one currently known original specimen of *P.neglectum* collected by Mönkemeyer (Wesergebirge: in Erlenbrüchen bei Eschershausen, Juli 1900, *Mönkemeyer s.n.*, B 30 0105646). However, like the other ones, this is also not mentioned in the protologue (Fig. 1B).

The characteristics given in the diagnosis (e.g., shrunken turf; strong costae; loose cell areolation) and figures (e.g., rather flat, symmetrical, ovate leaves; straight apex; short, wide, hexagonal, or narrowly hexagonal cells) are consistent with those of the *Stereodonnemoralis* Mitt. lectotype (Wolski et al. 2020). Only the leaf apex from the Mönkemeyer (1927) figure is not serrate, as should be in this case. Nevertheless, based on the other features given above, the proposal by Nyholm (1965), Ireland (1969), and Iwatsuki (1970) that *P.neglectum* is the plant currently understood as *P.nemorale* (syn. *P.sylvaticum*) appears correct.

Due to the existence of Figure 207c of this taxon, to which Mönkemeyer (1927) only refers to in the protologue, and according to Art. 9.1 of the *Shenzhen Code* (Turland et al. 2018), it should be assumed that Figure 207c is the holotype of that name. However, because the leaf apex of the holotype is not serrate, this suggests that the material used to make Figure 207c was derived from two different taxa (mixed material).

**Figure 3.**
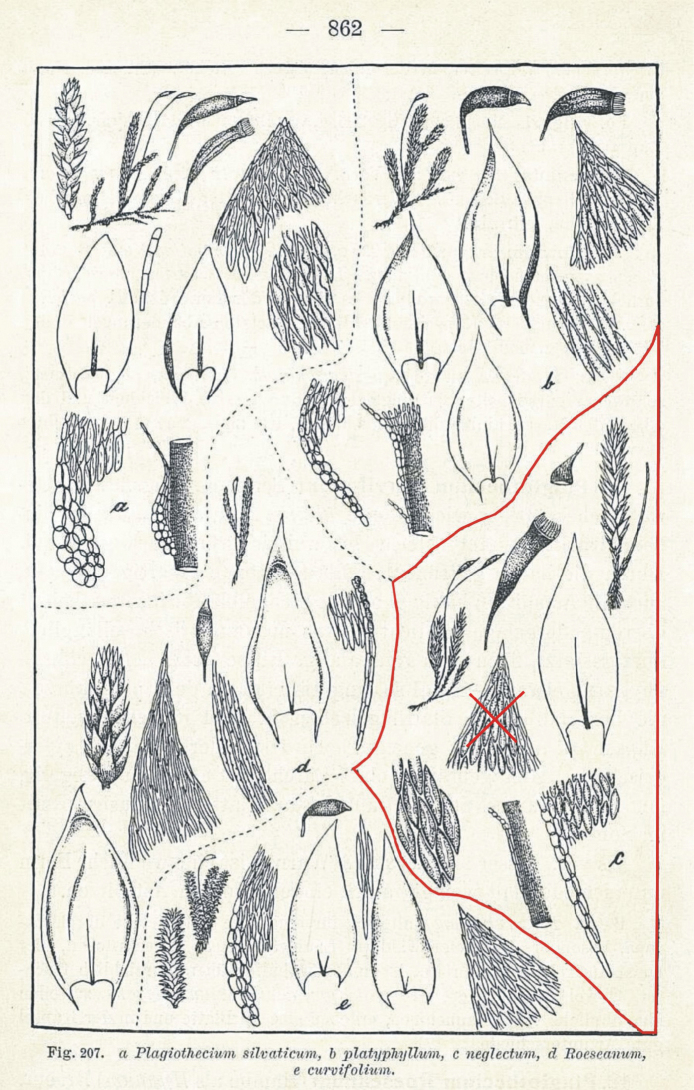
Lectotype of *Plagiotheciumneglectum* – outlined with a red line; the part of the figure showing the leaf apex is crossed out and excluded from the lectotype (figure 207c; Mönkemeyer 1927; changed).

According to Art. 9.3 of the *Shenzhen Code* (Turland et al. 2018), “A lectotype is one specimen or illustration designated from the original material (Art. 9.4) as the nomenclatural type, in conformity with Art. 9.11 and 9.12, (...) if a type is found to belong to more than one taxon (see also Art. 9.14)”. Therefore, the part of the figure representing the leaf apex, which is not serrate, should be excluded from the holotype, this being Fig. 207c (Mönkemeyer 1927: 862) (Fig. 3), and the rest of Figure 207c should be designated as the lectotype of *P.neglectum*.

**Figure 4.**
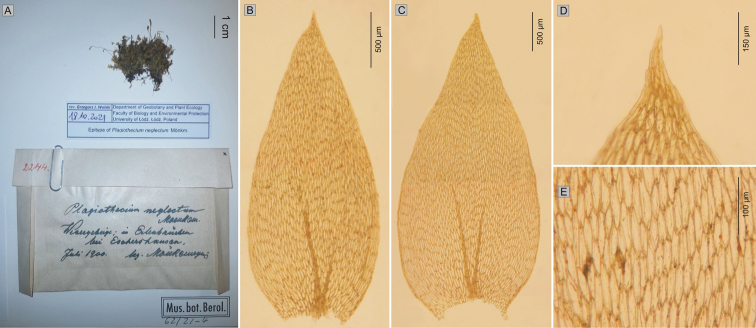
Epitype of *P.neglectum* and some of its most important taxonomic characteristics. **A** specimen from herbarium B (B 30 0105646) **B–C** stem leaves **D** serrate leaf apex **E** cells of the middle part of the leaf.

However, due to the fact that the newly designated lectotype does not include the figure of the leaf apex, which is an important taxonomic feature, a specimen recently found in Herbarium B (B 30 0105646) should be designated as the epitype; it is the original material (as stated in Article 9.4 of the *Shenzhen Code*), collected by Mönkemeyer and signed by him as “*P.neglectum*”. The plants of specimen B 30 0105646 also show a serrate leaf apex (Fig. 4), which indisputably indicates an association with *P.nemorale*.

This is an excellent choice because this specimen comes from the original Mönkemeyer collection; this way, it is practically impossible to change the understanding of the name *P.neglectum*. In addition, it is not possible to find the remaining original materials, which, however, are not quoted in the protologue of *P.neglectum* but listed in the Walter and Martienssen (1976) catalog.

## ﻿Taxonomic treatment

*Stereodonnemoralis* Mitt., J. Linn. Soc. Bot. Suppl. 1: 104 (1859) ≡ *Plagiotheciumnemorale* (Mitt.) A.Jaeger, Ber. St. Gall. Naturw. Ges. 1876–1877: 451 (1878) ≡ P.silvaticumvar.nemorale (Mitt.) Paris, Index Bryol.: 967 (1898). **Type citation**: *Hab.* In Himalayae orient. reg. temp., Sikkim, in monte Tonglo (ad radicem filicis cujus dam), *J. D. Hooker* ! ***Lectotype***: “Herb. Ind Or Hook. Fil. & Thomson *Stereodonnemorale* m. Hab. Sikkim, Tonglo Regio temp. Alt. – J. D. H.” – BM 1030713!: ***isolectotype***: NY 913349! = *P.neglectum* Mönk., Laubmoose Europas 866 (1927). ***Lectotype*** (*designated here*): Figure 207c without a part of the figure with the top of the leaf (Mönkemeyer 1927: 862), see Figure 3. ***Epitype*** (*designated here*): Wesergebirge: in Erlenbrüchen bei Eschershausen, Juli 1900, *Mönkemeyer s.n.*, B 30 0105646 (Figure 4).

The remaining original material according to Walter and Martienssen (1976) was confirmed to have been lost at HBG: Thüringen: Eisenach, Annatal, 26.7.1898, u. Wartburg, 2.5.1915 (*J. Bornmüller s.n.*); Wesergebirge: Bodenwerder, Königszinne, Juli 1901 (*W. Mönkemeyer s.n.*); Hessen, Rhön: Gr. Nallen, Juli 1906 (*W. Mönkemeyer s.n.*). Vogtland: Plauen, Triebtal, 25.07.1904 (*E. Stolle s.n.*); Bayern: Allgäu, Hinterstein, Sauwald, Aug. 1906, u. Regensburg, U-Lichtenwald, Schindelmacherhänge, Nov. 1906 (*I. Familler s.n.*); Prien/Chiemsee: 500 m, Juni 1911 (*T. Linder s.n.*); Mähren: Oppafall, Juli 1904 (*J. Podpěra s.n.*); Ostpreuβen: Labiau, Juli 1864 (*H. v. Klinggräff s.n.*); Kurland: Usmaitensee, Moritzholm, Mengwald, 3.8.1913 (*K. R. Kupffer s.n.*); sine loc. et dat. (*Wüstnei s.n. 380*).
